# Behavior of RC Beams Strengthened Using Steel-Wire–Carbon-Fiber-Reinforced Plates

**DOI:** 10.3390/ma13183996

**Published:** 2020-09-09

**Authors:** Wanxu Zhu, Mingxia Li, Heying Qin, Feng Fu, Fengrong Liu

**Affiliations:** 1 College of Civil Engineering and Architecture, Guilin University of Technology, Guilin 541004, China; zhuwanxu@vip.163.com (W.Z.); Mingxiali@glut.edu.cn (M.L.); heying.qin@glut.edu.cn (H.Q.); Fengrong.liu@glut.edu.cn (F.L.); 2 Collaborative Innovation Center for Exploration of Nonferrous Metal Deposits and Efficient Utilization of Resources, Guilin University of Technology, Guilin 541004, China; 3 Guangxi Key Laboratory of New Energy and Building Energy Saving, College of Civil Engineering and Architecture, Guilin University of Technology, Guilin 541004, China; 4 School of Mathematics, Computer Science & Engineering, City, University of London, London EC1V 0HB, UK

**Keywords:** prestress, steel-wire–carbon-fiber-reinforced plate, RC beam, strengthening effect, ductility

## Abstract

The steel-wire–carbon-fiber-reinforced plate (SCFR plate) is a relatively new strengthening technology for concrete structures. In this paper, a series of lateral impact tests on SCFR plates and conventional carbon-fiber-reinforced plates (CFR plates) were first performed, followed by tensile tests of both the SCFR plates and the CFR plates. It is found that the SCFR plates can provide the same level of tensile strength as CFR plates, whilst having evident advantages in terms of better ductility and lateral resistance. It is also found that increasing the amount of the steel wire can improve the lateral resistance of the SCFR plate. In addition, the SCFR plate shows the advantage of a reduction in lateral damage, which is commonly experienced by CFR plates during transportation, construction, and maintenance. In the second stage of the research, flexural tests of both SCFR and CFR plate-strengthened reinforced concrete (RC) beams were performed. The failure modes and crack patterns of the RC beams were investigated. Results show that the SCFR plate-strengthened beam exhibits enhanced ductility compared to that strengthened by traditional CFR plates, thereby enhancing the flexural capacity of the RC beams. On the basis of the test results, a formula is designed to predict the flexural capacity of SCFR plates; good agreement is achieved.

## 1. Introduction

The use of carbon-fiber-reinforced plates (CFR plates) is a strengthening technology for concrete structures which has been adopted in retrofitting projects over the past 25 years [1,2,3,4]. CFR plates have numerous advantages, including a high bearing capacity, strong corrosion resistance, reduced weight, and fast track construction. It has, therefore, become an important research topic. Experimental studies of CFR plate-strengthened beams were carried out by Alfarabi [5], Garden [6], and Peng et al. [7]. In each study, it was found that prestressing the CFR plate significantly increased the material’s cracking and yield load. Czaderski et al. [8] used prestressed CFR plates anchored by a gradient method to establish flexural reinforcement and strengthen prestressed concrete beams in a 17 m long bridge in southern Switzerland, with good results. Chen et al. [9] discovered a significant improvement in fatigue performance for RC beams strengthened by CFR plates when handling vehicle overload for a bridge. In another study, Ghafoori et al. [10] looked at the crack propagation in steel beams strengthened by non-prestressed and prestressed CFR plates under cyclic loads and established a model for calculating the fatigue performance of strengthened beams on the basis of fracture mechanics. Together, these studies promoted the application of CFR plate strengthening technology in engineering and its progress.

CFR plate strengthening is now included in amended reinforcement design standards [11], and product standards now include performance requirements for carbon-fiber anchors [12]. As a result, the uptake of CFR plates in highway and railway engineering projects for the strengthening of concrete beams has risen dramatically. However, this has also revealed a number of problems. Firstly, the CFR plate is a brittle material [13]. If there is an imperfection in the prestressed system, the plate can suddenly facture under stretch during construction. Secondly, the transverse damage ability of the CFR plate is poor, and reliable anchoring is difficult [14,15]. Reinforcement failure cases are gradually occurring, and the safety risks are extensive. Thirdly, when a reinforced concrete (RC) beam is strengthened using a CFR plate, especially a prestressed CFR plate, its ductility is notably reduced. Lastly, in the process of transportation and construction, carbon-fiber board is easily damaged. Although the damaged carbon-fiber board does not fail in the process of tension, it suddenly breaks due to the accumulation of damage during the service period, and the consequences are unimaginable [16,17].

The CFR plate is prone to damage during the transportation and the construction processes. Even if damage of a CFR plate has not yet occurred, sudden fractures due to accumulated damage during transportation may cause serious consequences. In this paper, through detailed experimental tests, we evaluate the possibilities of using a steel-wire–carbon-fiber-reinforced plate (SCFR plate) to overcome the brittle characteristics and poor lateral shear resistance of CFR plates.

Recently, composite materials such as CFR plates with embedded steel wire [18,19,20,21] have attracted substantial research interest. The uniaxial tensile properties of hybrid fiber composite materials were studied by Young et al. [22], as well as the effect of different parameters, such as fiber type, amount of carbon fiber, and paving mode on material performance. The results of this work allowed determining the uniaxial tensile elastic modulus of hybrid composite materials. Luo et al [23] put forward a steel continuous fiber composite reinforcement (SFCB) made of steel and fiber. On the basis of a large number of tests, the ideal state of the structure of SFCB was explored; the material properties and production process of each component of SFCB in industrial preparation were introduced in detail; the performance and price of the SFCB, steel bar, and FRP bar were compared, which proved that SFCB is obviously superior to the FRP bar in terms of mechanical properties and price. Wu et al. [24] conducted monotonic tensile tests of self-made steel wire continuous basalt fiber composite plates and discovered that the specimens had good mechanical properties and could continue to withstand large loads after some of the fibers had fractured. This enhanced the component ductility. In the field of composite materials for aerospace, research focused on lateral damage and impact resistance [25,26]. Finite element approaches were also used to simulate the low-speed impact failure of carbon-fiber composite plates and their residual stress after impact loading [27,28], with the outcomes of these simulations being subjected to experimental verification. RC beams strengthened with SCFRP can be widely used in civil engineering; for example, in tall buildings [29,30], they can be used to strengthen the connection [31] or frames [32,33] to avoid progressive collapse. They can also be used in bridge engineering. However, research on this new type of strengthening technique is limited.

In this paper, a series of tests, including lateral impact tests, on the SCFR plate were first performed. It is found that the SCFR plate can provide the same level of tensile strength as the carbon-fiber-reinforced plate (CFR plate), whilst having evident advantages in terms of better ductility and lateral resistance. An increase in the amount of the steel wire can improve the lateral resistance of the SCFR plate. In addition, the SCFR plate can reduce the lateral damage-induced failure commonly encountered during transportation, construction, and maintenance. In the second stage of the test, RC beams strengthened by means of prestressed SCFR plates were also tested. Results show a significant improvement in the flexural capacity of the RC beam. It also offers a way of dealing with the problem of insufficient ductility when using a conventional CFR plate to improve the bearing capacity of RC beams.

In order to improve the transverse shear capacity of carbon-fiber-reinforced plates (CFR plates), reduce the cost, and retain the advantages of the original reinforcement technology, a new type of embedded steel-wire–carbon-fiber board (SCFR plate) is proposed by using high-strength steel wire and carbon-fiber pultrusion technology. The new composite has the same mechanical properties as pure carbon-fiber board. While retaining the advantages of pure carbon-fiber board, it improves the transverse shear performance of carbon-fiber board and provides more effective protection in the whole process of carbon-fiber board reinforcement. In order to provide technical support for the engineering application of SCFR plates, this paper carries out mechanical property tests and reinforced RC beam tests, aiming to provide technical support for the engineering application of SCFR plates.

## 2. Impact and Tensile Tests on SCFR Plates and CFR Plates

To study the performance of SCFR plates, a series of tests was performed as explained in this section. In the meantime, the same tests were performed on CFR plates as a comparative study.

**Figure 1:**
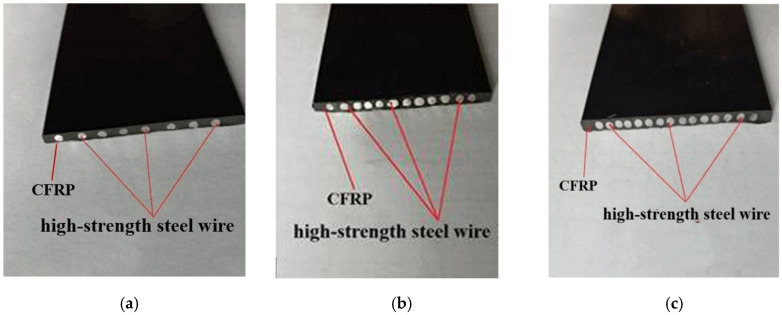
Steel-wire–carbon-fiber-reinforced plates. (**a**) SCFR Plate-1; (**b**) SCFR Plate-2; (**c**) SCFR Plate-3.

The quantity, diameter, and arrangement of the high-strength steel wires in the carbon fiber were designed according to the basic parameters shown in Table 1. During the production, the position of steel wires in the plates coincided the carbon fibers at corresponding points. The high-strength steel wires and carbon-fiber wires were then pulled together by external force along the FRP plate, after passing through a resin dip tank. After this, the wire and fiber plates were formed by heat extrusion using molds. The CFR plate and SCFR plate cross-sectional dimensions used in the tests were 50 mm × 3 mm. The steel wire content in the SCFR PLATE was varied according to specimen as 0%, 16.75%, 25.12%, or 31.4%. Pictures of the actual products are shown in Figure 1.

### 2.1. Low-Speed Lateral Impact Tests on SCFR and CFR Plates

**Figure 2:**
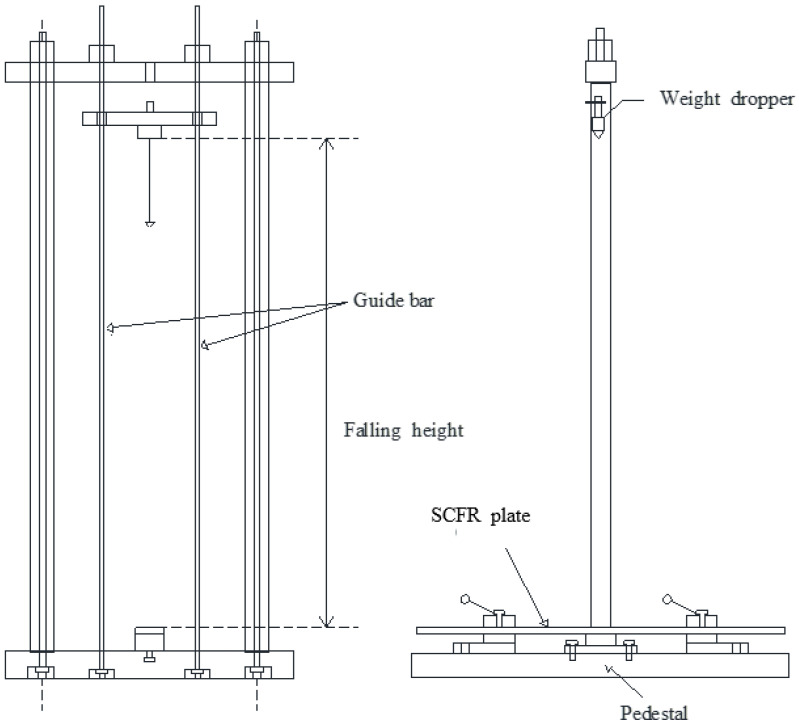
The drop hammer impact device.

In this study, a drop hammer impact device was specifically designed to conduct lateral impact tests on these two types of plates (as shown in Figure 2). The CFR plate specimens were placed on the pedestal as shown in Figure 2. To ensure very little bending deformation after lateral impact, the specimen was fixed on by pressing sheets. The hammer’s position and the central position of the CFR plate were kept consistent. The strain energy due to bending deformation was, therefore, ignored, and all the impact energy was presumed to be directly converted into kinetic energy, causing the destruction of the material, which was characterized by fracture and extrusion failure of the fiber bundles and resin matrix. The test results show the changed noticeable damage due to impact energy.

In total, six CFR and SCFR plates were tested, as outlined in Table 1, and trial tests were carried out before formal testing. The travel distance of the hammer ranged from 100 mm to 600 mm. The impact load was changed by adjusting the drop height of the hammer, with an initial impact height of 100 mm. During the tests, the impact height was increased by increments of 100 mm until 600 mm. Six measuring points were made with equal distance through the depth of damage grooves. The fracture of the fibers on both sides of the specimens was monitored during the tests.

**Figure 3:**
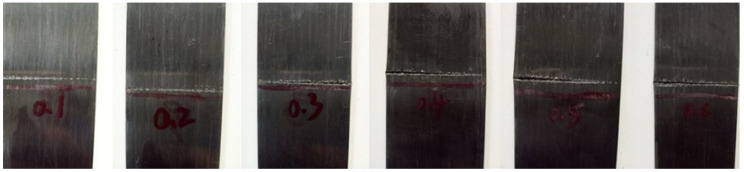
Damaged specimens.

When the impact height was 100 mm, light grooves began to appear on the surface of the specimens. When the impact height was 200 mm, some fibers in the specimens were fractured and evident grooves appeared, but there were no evident impact marks on the back. When the impact height was increased to 300 mm and 400 mm, the steel wire in the grooves was exposed and the groove depth increased. When the impact height reached 500 mm and 600 mm, a large number of carbon fibers in the specimen were fractured and warped upward, showing deep grooves and longitudinal cracks. The back of the specimens also showed a slight outward bulge. Some damaged specimens are shown in Figure 3. By comparing the residual tensile strength of the specimens with different drop heights, it can be noticed that, when the travel height of the hammer was 600 mm, the residual strength was about 70% of the original strength.

**Figure 4:**
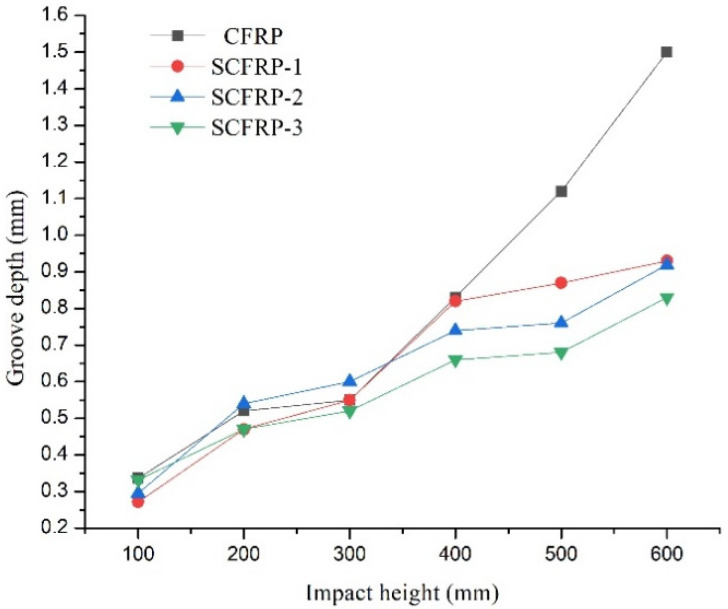
Groove damage depth/impact height curves for the (S)CFR plate specimens.

The relationship between the groove damage depth of the specimens and the impact height is explored in Figure 4. It can be seen that, when the impact height was increased from 200 mm to 300 mm, the groove depth of each specimen varied from 0.52 mm, 0.47 mm, 0.54 mm, and 0.47 mm to 0.55 mm, 0.55 mm, 0.6 mm, and 0.52 mm, respectively, with the corresponding rates of increase of the groove depth being 5.4%, 17%, 7.4%, and 10.6% respectively. The change in groove depth for each specimen was less than 0.1 mm, and in no case did the groove depth reach the surface of the steel wires when the impact height was less than 300 mm; thus, the steel wires had little effect upon the depth of damage. After the impact height rose above 300 mm, the groove depth was noticeably increased. When the impact height changed from 300 mm to 600 mm, in steps of 100 mm, the groove depth for the CFR PLATE specimens increased from 0.55 mm to 0.83 mm, 1.12 mm, and 1.5 mm, with a corresponding rate of increase of 50.9%, 103.4%, and 172.7%, respectively. For the SCFR Plate-1 specimen, the groove depth increased from 0.55 mm to 0.82 mm, 0.87 mm, and 0.93 mm, with a corresponding rate of increase of 49.1%, 58.2%, and 69.1%, respectively. For SCFR Plate-2, the groove depth increased from 0.6 mm to 0.74 mm, 0.76 mm, and 0.92 mm, with a corresponding rate of increase of 23.3%, 26.7%, and 53.3%, respectively. For SCFR Plate-3, the groove depth increased from 0.52 mm to 0.66 mm, 0.68 mm, and 0.83 mm, with a corresponding rate of increase of 26.9%, 30.8%, and 59.6%, respectively. It can be concluded from these results that, when the impact height was greater than 300 mm, the depth of the grooves reached the surface of the steel wires, and they started to provide resistance to lateral damage. As there were no steel wires in the CFR plate specimens, the groove depth increased rapidly due to the fact that no resistance of the steel wire existed. For the SCFR plate specimens, however, as the amount of steel wire increased, the increase in the groove depth became slower when the impact height increased. This suggests that a higher steel wire content results in greater ability of the SCFR plate specimens to resist lateral damage.

### 2.2. Tensile Tests of SCFR and CFR Plates 

**Figure 5:**
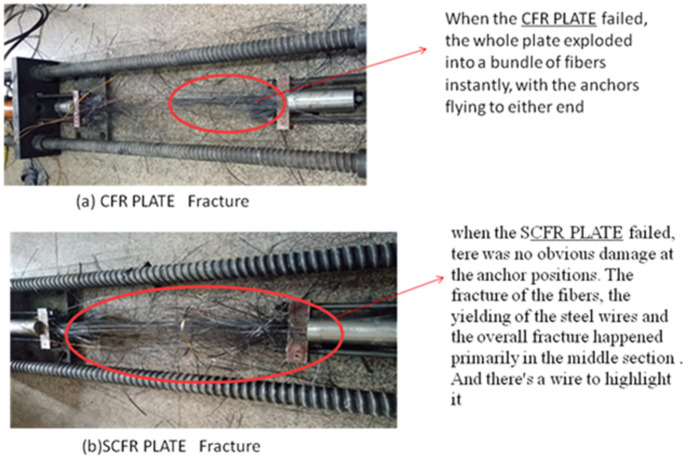
(**a**) CFR plate fracture; (**b**) SCFR plate fracture.

**Figure 6:**
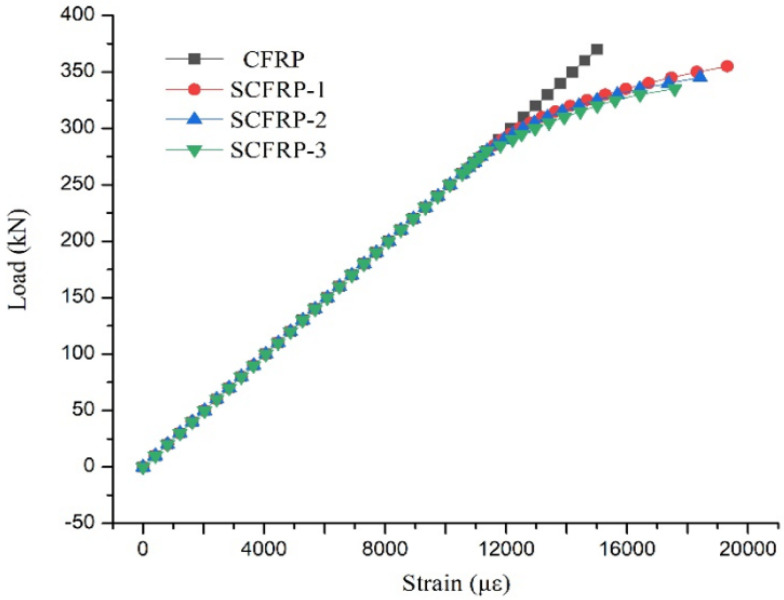
Load–strain relationship curves for the CFR plate and SCFR plate specimens.

The CFR plate and SCFR plate specimens, shown in Table 1, were tested using traditional tensile test rigs available at the Guilin University of Technology. From the tests, it was found that the steel and carbon-fiber wires proved to be well bonded, with no stripping phenomenon occurring during the tests. Different failure modes were observed for these two types of specimens. When the CFR plate failed, the whole plate instantly exploded into a bundle of fibers, with the anchors flying to either end, as shown in Figure 5a. In contrast, when the SCFR plate failed, there was no obvious damage at the anchor positions. The fracture of the fibers, the yielding of the steel wires, and the overall fracture happened primarily in the middle section, as shown in Figure 5b. The high-strength steel wires showed good resistance to deformation and were able to absorb the strain energy released by the broken carbon fibers after their fracture. As can be seen from Figure 6, there was no obvious yield point for the CFR plate specimens, and the load–strain relationship changed linearly up to ultimate tensile force of 370 kN. In contrast, the load–strain curves for the three SCFR plate specimens showed an obvious yield point, at about 280 kN, with an ultimate tensile strength of 355 kN, 345 kN, and 335 kN, representing 95.9%, 93.2%, and 90.6% of the CFR PLATE value, respectively. The decrease in strength limit was not significant, with the difference being less than 10%. Plastic deformation was observed in SCFR plate specimens. The ultimate strain of specimens SCFR Plate-1, SCFR Plate-2, and SCFR Plate-3 further increased by 4000 με, 3000 με, and 2000 με after yield, respectively, and their plastic strain rates increased by 17.5%, 20.9%, and 29.4%. This shows that, under the same tensile force, more steel wire content in the SCFR plate leads to greater strain increase.

### 2.3. Tensile Tests of SCFR and CFR Plates after Impact Tests

**Figure 7:**
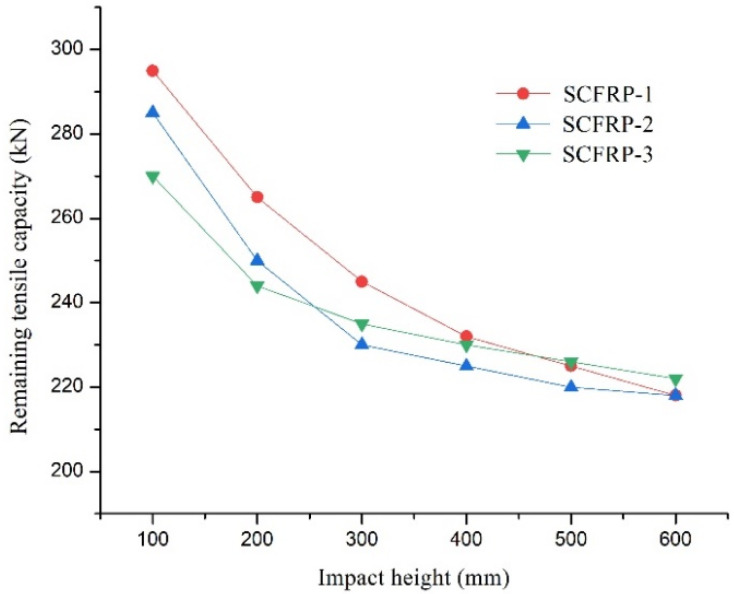
Residual tensile capacity/impact height curves for the SCFR plate specimens.

After impact testing, further tensile tests of SCFR and CFR plates, damaged by lateral impact, were carried out to determine their residual tensile capacity. The test results are shown in Figure 7.

It can be seen from Figure 7 that the residual tensile capacity of the SCFR plate specimens decreased gradually as the impact height increased. When the impact height increased from 100 mm to 300 mm, the residual tensile capacity of specimens SCFR Plate-1, SCFR Plate-2, and SCFR Plate-3 decreased from 295 kN, 285 kN, and 270 kN to 245 kN, 230 kN, and 235 kN, respectively, with the corresponding rates of decrease being 16.9%, 19.2%, and 12.9%, respectively. At this stage, the damage did not extend to the steel wires; thus, the effect of the steel wire content on the change in residual tensile capacity was limited in these conditions. When the impact height increased from 300 mm to 600 mm, the steel wire began to experience damage and the residual tensile capacity of specimens SCFR Plate-1, SCFR Plate-2, and SCFR Plate-3 was decreased to 218 kN, 218 kN, and 222 kN, respectively. The corresponding rate of decrease in tensile capacity was 11%, 5.2%, and 5.5% respectively, which again indicates that a higher steel wire content at this stage had a lower effect on the material’s residual tensile capacity.

## 3. Experimental Study of RC Beams Strengthened by (S)CFR Plates

### 3.1. Test Specimens

The strengthened concrete beams had a rectangular cross section of b × h = 250 mm × 400 mm and a beam span of 4200 mm. The clear span was 4000 mm, and the concrete’s designed strength was C40. The reinforcement of the specimen was as follows: two Φ 20hrb335 tensile longitudinal bars were set at the bottom, two Φ 10hpb300 vertical bars were set in the upper compression area according to the structure, and the stirrups were distributed, whereas the whole beam was Φ 20hrb335 10@120 mm. The thickness of the concrete cover was 25 mm.

**Figure 8:**
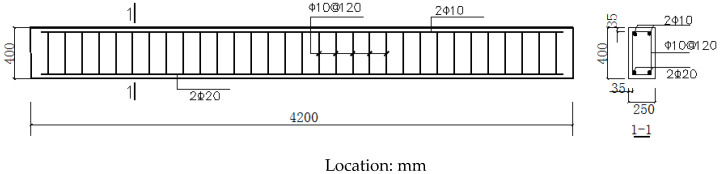
Detailing of reinforcement for reinforced concrete (RC) beam.

Altogether, six tests were performed, with two CFR plate-strengthened beams, three SCFR plate-strengthened beams, and one reference beam without any strengthening (as explained in Table 2). Among them, some involved CL1 and ZSCL-1 to 3 using prestressed (S)CFR plate-strengthening techniques. CL-0 used the conventional CFR plate-strengthening technique. The cross-sectional size and length of the (S)CFR plates used for strengthening was 50 mm × 3 mm and 3000 mm. The specific dimensions and reinforcement of the specimens are shown in Figure 8, and their basic parameters are provided in Table 2.

**Figure 9:**
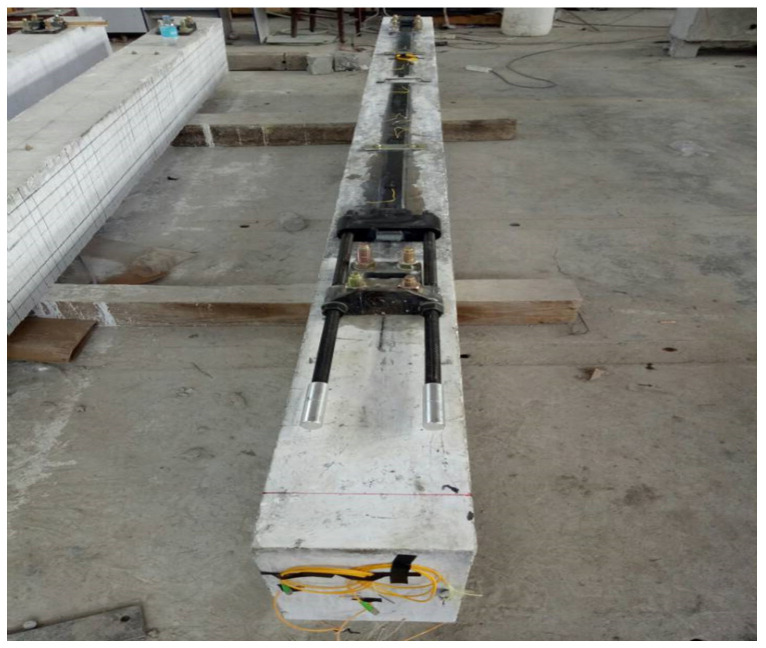
An RC beam specimen after installation of the prestressed SCFR plate.

**Figure 10:**
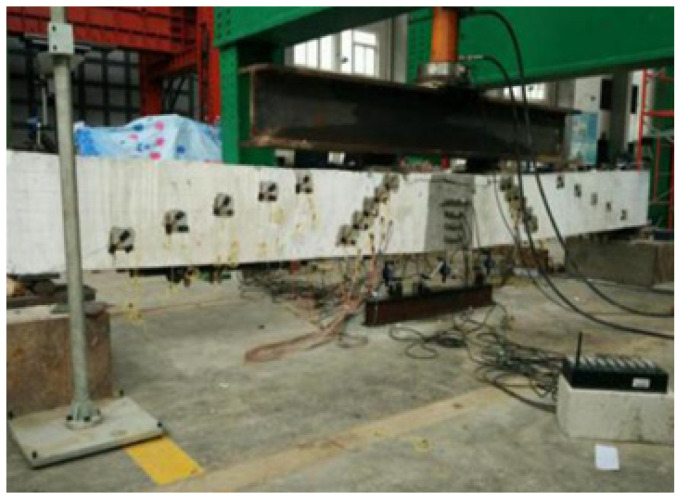
The loading process.

After the concrete beam specimen was made, a water drill was used to drill holes in the corresponding anchorage points at the bottom of the concrete beam, and the anchoring support was fixed on the bottom surface of the concrete beam with a chemical anchor bolt, while the anchored SCFR plate was clamped on the support seat. After ensuring that there was no cavity, the structure glue was filled between the carbon-fiber board and the bottom of the beam, and the pressure strip was fixed on the carbon plate. The structural adhesive was installed 500 mm away from the anchorage at both ends, resulting in the SCFR plate being close to the bottom of the beam; the surplus carbon board glue was cleaned up. The method of step loading was used to apply prestressing force to the SCFR plate. After loading, the load was held for 5 min. The bolts were tightened to maintain the prestress, and the sensors and jacks were removed. The fiber grating was pasted in the groove with adhesive under the loading state of carbon-fiber board. After the installation of prestressed SCFR plates, the static load test of the specimens was carried out with the loading system. A two-point symmetrical loading mode was adopted in the test, and the distance between loading points was taken as 800 mm. A fiber Bragg grating was coupled with the prestressed carbon-fiber board, and its strain was measured using a fiber grating demodulator. The specimen of a prestressed SCFR plate after installation is shown in Figure 9, and the field loading diagram is shown in Figure 10.

### 3.2. Instrumentation and Loading Process

The strain of the longitudinal reinforcement, the strain of concrete and the deflection of the beam, the development of cracks, and the slip were monitored during the tests using similar instrumentation to [34,35,36,37,38].

A four-point bending test was carried out through the distribution beam (as shown in Figure 10). The loading process was as follows: first, the theoretically calculated cracking load was preloaded to 40%, all instruments were verified to be normal before returning to zero, and then the formal loading was started. Loading was increased by 10 kN/level (5 kN/level) until the test beam had cracks. After the crack was formed, 70% of the theoretical yield load was taken as the critical value. Before reaching the critical value, a loading speed of 20 kN/level was used. After reaching this critical point, a loading speed of 10 kN/level was used until the tensile longitudinal bar at the bottom of the beam yielded. After yielding, load control was no longer used, and the loading rate was 2 mm/grade until the concrete in the compression zone was crushed or the CFR plate was broken.

### 3.3. Discussion

The test results show that, when the beams reached their limit capacity, the tensile bars yielded, but there was no bond failure between the prestressed (S)CFR plate and the concrete. In view of this, the stress block for the compressed section of the component can be simplified to an equivalent rectangular stress block. As the pre-generated tensile strain in the tensile region caused by the prestress can lead to strain hysteresis in the (S)CFR plate, the effect of the prestress on the ultimate flexural bearing capacity is limited and can be ignored in the calculations.

### 3.4. Formula to Calculate the Flexural Capacity

From the equilibrium equations, the following can be obtained:

(1) $$M=\alpha_{1}f_{c0}bx(h_{0}-x/2)$$

(2) $$\alpha_{1}f_{c0}bx=f_{f}A_{f}+f_{y0}A_{s0},$$

(3) $$2a^{\prime }\leq x\leq \xi_{b,f}h_{0},$$

where *h*_0_ is the effective height of the section before reinforcement of the component, *ξ_b,f_* is the ratio of the height of the compressive zone to the effective height in the strengthened flexural member, taken as 0.85 times the control value before strengthening, *M* is the ultimate bending bearing capacity, *α_1_* is the calculation coefficient, *f_c_*_0_ is the designed value of compressive strength in the concrete axis, *x* is the height of the compressive zone of the concrete, *b* and *h* are the width and height of the rectangular section, respectively, *f_y_*_0_ is the designed value for the tensile strength of the tensile bar, *A_s_*_0_ is the cross-sectional area of the tensile bar, *f_f_* is the designed value for the tensile strength of the (S)CFR plate, and *A_f_* is the cross-sectional area of the (S)CFR plate.

When the test beams were strengthened, because of the large tensile strain of the (S)CFR plate specimens, the height of the compressive zone of the concrete was small. Thus, the contribution of the concrete in the compressive zone can be ignored during the calculations. The stress block in the compressive zone also had little effect on the ultimate flexural capacity, allowing it to be simplified to an equivalent rectangular stress block. This could be obtained using the following section balance equations:

(4) $$M=f_{y0}A_{s0}(h_{0}-x/2)+\sigma_{f}A_{f}(h-x/2)$$

(5) $$\alpha_{1}f_{c0}bx=f_{y0}A_{s0}+\sigma_{f}A_{f},$$

where *A_f_* is the cross-sectional area of the (S)CFR plate, *σ_f_* is the tensile stress of the (S)CFR plate, *h* is the distance between the center of the (S)CFR plate and the edge of the compressive zone of the concrete, and *M* is the bending bearing capacity of the positive cross-section.

For CL-0 without prestress, the initial strain of the (S)CFR plate was 0 and the steel bar yielded, whereas the concrete reached its strain limit at the breaking point. This could be obtained according to the assumption that the section remains as a plane.

(6) $$\varepsilon_{f}=\frac{h-x}{x}\varepsilon_{cu}$$

From the equilibrium equations, one can obtain the following:

(7) $$=f_{y0}A_{s0}\left(h_{0}-\frac{x}{2}\right)+\varepsilon_{f}E_{f}A_{f}\left(h-\frac{x}{2}\right),$$

(8) $$\alpha_{1}f_{c0}bx=f_{y0}A_{s0}+\varepsilon_{f}E_{f}A_{f},$$

where *ε_f_* is the strain of the (S)CFR plate at breaking point, *E_f_* is the elastic modulus of the (S)CFR plate, *h* is the distance between the center of the (S)CFR PLATE and the edge of the compressive zone of the concrete, and *M* is the bending bearing capacity of the positive cross-section.

### 3.5. Calculation Formula Validation

Using Equations (1)–(8), the flexural capacity of each test beam can be calculated. The calculation results are shown in Table 3 and compared with the test results. 

From Table 3, it can be seen that the test results of each group of beams in the experiment were in good agreement with the calculated values. The mean error for the ordinary prestressed (S)CFR plate was 3%, and the mean error for the prestressed reinforced (S)CFR plate was 3.6%.

Using the cracking load and ultimate load of each beam, measured in the experiment, the cracking bending moment and the ultimate bending moment of each tested beam were also calculated. The results are shown in Table 4.

In Table 4, a comparison of L-1, CL-0, and CL-1 reveals that the cracking load, yield load, and ultimate load of the reinforced components were enhanced by strengthening them with the CFR plate. Prestressing made the reinforcement effect even more evident. Comparing the results for the reference beam L-1 with the cracking load for the beams reinforced with SCFR plates (ZSCL-1, ZSCL-2, and ZSCL-3), the capacity was improved by 293.3%, 291.8%, and 291.8%, respectively. The cracking load for the beam reinforced by the CFR plate, CL-1, was improved by 291.8%, which is consistent with the above values, but also noticeably higher than the improvement coefficient for the beam without any prestress, CL-0 (194.7%). For the yield load, the improvement coefficients for the beams reinforced by SCFR plates were 200%, 238.5%, and 226.5%, respectively, which were very close to the improvement coefficient for the CFR plate-reinforced beam, CL-1 (218.2%), but also higher than the improvement coefficient for CL-0 (154.5%). The average value for the ultimate load for the three groups of components reinforced by SCFR plates was 285 KN, which was basically the same as the value for the component reinforced by the CFR plate (282.4 KN). When compared to the reference beam L-1, the ultimate load for the SCFR plate-reinforced beams was improved by 197.1%, 211.6%, and 219.2%, respectively, whereas, for CL-1, the improvement was 206.1%. These values were close to each other, but also higher than the improvement coefficient for CL-0 without prestress (153.3%). On the basis of the above analysis, it is clear that prestressed SCFR plates can significantly improve the cracking load, yield load, and ultimate load of an RC beam. At the same time, when compared to a beam reinforced using a pure CFR plate, the addition of steel wire and the decrease in carbon-fiber content do not change the strengthening effect.

## 4. Deformation and Ductility 

The mid-span deflection values for the specimen are shown in Table 5.

**Figure 11:**
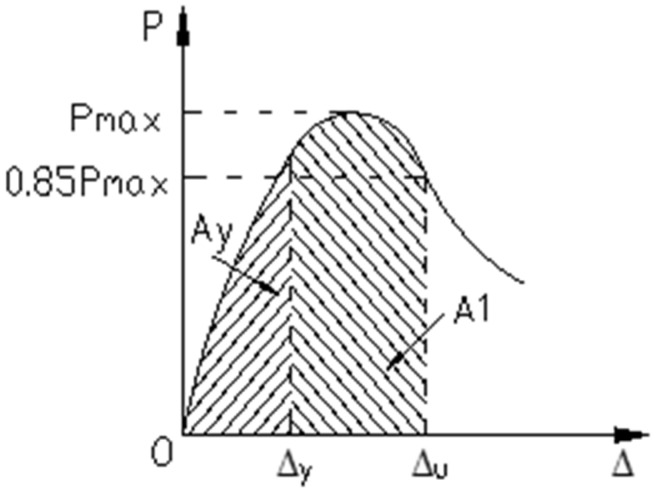
Defining the ductility factor.

**Figure 12:**
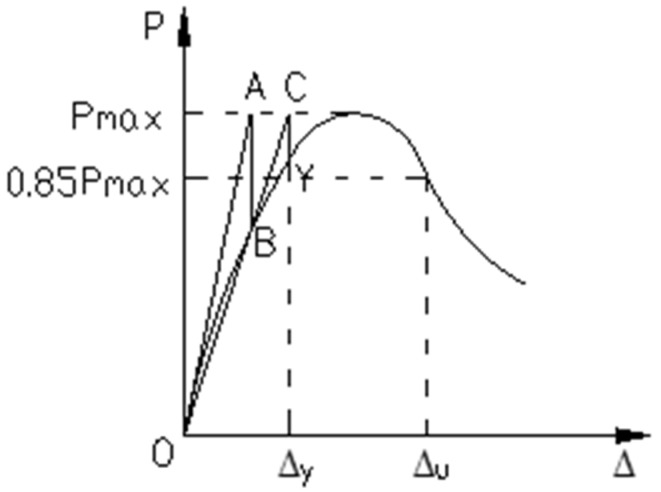
Determining the initial yield point.

In order to measure and compare the ductility of the five components, a ductility factor was taken as the measurement index, using the following formulas:

(9) $$\mu_{1}=\frac{\Delta_{u}}{\Delta_{y}},$$

(10) $$\mu_{2}=\frac{A_{u}}{A_{y}},$$

where Δ*_u_* is the deflection at the point where the load is 0.85*P_max_* in the downward section of the load–deflection curve, Δ*_y_* is the deflection corresponding to the yield point Y of the beam, *A_y_* is the area contained by the load–deflection curve and the displacement axis between the origin and the initial yield point Y, and *A_u_* is the area contained by the load–deflection curve and the displacement axis between the origin and the point corresponding to 0.85*P_max_* in the downward section of the load–deflection curve, as shown in Figure 11. *A_u_* = *A_y_* + *A*_1_. The initial yield point Y can be determined using geometrographic methods. The tangent line OA of the initial segment of the curve at the origin point intersects with the horizontal straight line passing through the load limit at point A. The vertical line passing through A parallel to the Y axis intersects with the curve at point B. Points O and B are connected, and this line intersects with the horizontal straight line passing through the load limit at point C. A line vertical to the X axis is drawn through point C, which intersects with the curve at point Y, which was the initial yield point. This process is shown in Figure 11 and Figure 12.

Calculated using Equations (9) and (10) and the test data, the ductility coefficients of the tested beams are shown in Table 6.

From the data in Table 5 and Table 6, it can be seen that, in the initial stage, the deformation and flexural capacity of the beams decreased and, while the steel wire was in its elastic stage, there was no difference in the deformation of the two types of strengthened beams. In the latter stage, the ductility of the five strengthened beams was significantly smaller than that of the refence beam. For *μ*_1_, the reduction was 15.4%, 30.7%, 19.2%, 15.4%, and 26.9%, respectively. For *μ*_2_, the reduction was 11.9%, 38.3%, 24.8%, 25.0%, and 31.2%, respectively. The reduction in the ductility factor for the prestressed strengthened reinforced beams was noticeably greater than that for the non-prestressed strengthened beams. When compared to the beams reinforced by the CFR plate without steel wire, the ductility of the beams reinforced by SCFR plates was obviously better, with an improvement for *μ*_1_ of 16.7%, 22.2%, and 5.6%, respectively, and for *μ*_2_ of 22.1%, 21.7%, and 11.6%, respectively. Thus, for the concrete beams strengthened with steel-wire–carbon-fiber plate, the ductility was greatly enhanced compared to the more commonly used pure CFR plate-strengthened concrete beams.

## 5. Conclusions

This paper reported results of a series of tensile and lateral low-speed impact tests using both CFR and SCFR plates. (S)CFR plate-strengthened RC beams were then tested to investigate their basic mechanical properties and the strengthening effect of various forms of (S)CFR plate. The conclusions are as follows:

(1) In the tensile tests, the steel wire had good deformation performance and could absorb the strain energy released by the broken carbon fibers, providing an initial buffer for the failure process. When the CFR plate and SCFR plate specimens were damaged, the pure CFR plate specimens burst into bundles of fibers and the anchors were pulled out. The SCFR plate specimens, by contrast, showed no slip phenomenon and the integral fracturing was concentrated in the middle section, with no obvious damage around the anchors.
(2) In the low-speed lateral impact tests and tensile tests after damage, when the impact height was less than 300 mm, the amount of steel wire had little effect on the depth of damage or remaining tensile capacity. When the impact height exceeded 300 mm, as the steel wire amount increased, the extent of damage reduced when impact height increased.
(3) In tests conducted with the (S)CFR plate-reinforced concrete beams, the flexural capacity of the beams reinforced with prestressed SCFR plates was close to that of those reinforced with the prestressed CFR plate. Thus, replacing some of the carbon fiber in the CFR plate with high-strength steel wire did not reduce the strengthening effect.
(4) The ductility of the beams could be reduced when strengthened with the prestressed (S)CFR plates, but the ductility for the prestressed SCFR plates was notably better than the commonly used CFR plate.
(5) Using SCFR plates to replace the conventional CFR plate for strengthening RC beams can further improve the flexural capacity, together with improved ductility. This makes the use of this form of reinforcement in engineering applications promising, whilst reducing the potential safety risks during the transportation of CFR plate-reinforced beams or their use in construction and service.
(6) Prestressed SCFR plates can significantly increase the crack load, yield load, and ultimate load of RC beams, compared with the pure CFR plate, whereas the addition of steel wire and the reduction of carbon-fiber content in the SCFR plates did not change their strengthening effect, while they provided an improvement in the bearing capacity.

## Figures and Tables

**Table 1:** Basic composition parameters of carbon-fiber-reinforced (CFR) steel-wire–carbon-fiber-reinforced (SCFR) plates.

(S)CFR Plate Specimen	Quantity and Diameter of Steel Wire	Volume of Steel Wire (%)	Volume of Carbon Fiber (%)
CFR Plate	0	0.00	70
SCFR Plate-1	8Φ2	16.75	53.25
SCFR Plate-2	12Φ2	25.12	44.88
SCFR Plate-3	15Φ2	31.4	38.6

**Table 2:** Specimen parameters.

No.	Strengthening Mode	Volume of Steel Wire (%)	Initial Stress Level (MPa)
L-1	Reference beam	0.0	-
CL-0	CFR PLATE	0.0	0
CL-1	CFR PLATE	0.0	1200
ZSCL-1	SCFR PLATE	16.75	1200
ZSCL-2	SCFR PLATE	16.75	1200
ZSCL-3	SCFR PLATE	16.75	1200

**Table 3:** Comparison between the calculated flexural capacity and the test results.

Specimen No.	The Loading $\frac{F^{T}}{kN}$	The Tested Bending Moment * $\frac{M^{T}}{kN\cdot m}$	The Calculated Flexural Capacity $\frac{M^{C}}{kN\cdot m}$	$\frac{M^{C}}{M^{T}}$
L-1	137	109.60	97.3	0.89
CL-0	210	168.00	142.41	0.85
CL-1	282.4	225.92	219.3	0.97
ZSCL-1	270	216.00	214.6	0.99
ZSCL-2	289.9	231.92	214.6	0.93
ZSCL-3	300.2	240.16	214.9	0.89

*: weight of specimens and equipment is not taken into account.

**Table 4:** Experimental results.

Specimen No.	Cracking Load (kN)	Improvement Coefficient (%)	Yield Load (kN)	Improvement Coefficient (%)	Ultimate Load (kN)	Improvement Coefficient (%)	Concrete Condition in the Compressive Zone
L-1	20.8	-	110	-	137	-	Crushed
CL-0	40.5	194.7%	170	154.5%	210	153.3%	Crushed
CL-1	60.7	291.8%	240	218.2%	282.4	206.1%	Crushed
ZSCL-1	61	293.3%	220	200%	270	197.1%	Crushed
ZSCL-2	60.7	291.8%	262.3	238.5%	289.9	211.6%	Crushed
ZSCL-3	60.7	291.8%	249.2	226.5%	300.2	219.2%	Tensile failure of carbon plate

**Table 5:** Comparison of the mid-span deflection values for the specimens.

Specimen No.	Mid-Span Deflection under Cracking Load (mm)	Mid-Span Deflection under an 80 KN Load (mm)	Mid-Span Deflection under a 200 KN Load (mm)	Mid-Span Deflection under Yield Load (mm)	Maximum Deflection (mm)
L-1	1	10.46	-	15.93	41.58
CL-0	2.03	9.35	-	16.83	39.83
CL-1	2.64	3.39	12.93	17.43	31.63
ZSCL-1	3.01	3.94	15.47	17.65	42.6
ZSCL-2	3.46	4.52	13.48	20.01	46.86
ZSCL-3	3.81	4.8	15.41	19.95	37.53

**Table 6:** The ductility factor of the components.

Specimen No.	Maximum Deflection (mm)	Ductility Factor $\mu_{1}$	$\frac{\mu_{1\left(x\right)}-\mu_{1}{}_{\left(L-1\right)}}{\mu_{1}{}_{\left(L-1\right)}}$	$\frac{\mu_{1\left(x\right)}-\mu_{1}{}_{\left(CL-1\right)}}{\mu_{1}{}_{\left(CL-1\right)}}$	Ductility Factor $\mu_{2}$	$\frac{\mu_{2\left(x\right)}-\mu_{2}{}_{\left(L-1\right)}}{\mu_{2}{}_{\left(L-1\right)}}$	$\frac{\mu_{2\left(x\right)}-\mu_{2}{}_{\left(CL-1\right)}}{\mu_{2}{}_{\left(CL-1\right)}}$
L-1	41.58	2.6	-	-	4.04	-	-
CL-0	39.83	2.2	−15.4%	-	3.56	−11.9%	-
CL-1	31.63	1.8	−30.8%	-	2.49	−38.4%	-
SCL-1	42.6	2.1	−19.2%	16.7%	3.04	−24.8%	22.1%
SCL-2	46.86	2.2	−15.4%	22.2%	3.03	−25.0%	21.7%
SCL-3	37.53	1.9	−26.9%	5.6%	2.78	−31.2%	11.6%
